# Deep learning for detecting visually impaired cataracts using fundus images

**DOI:** 10.3389/fcell.2023.1197239

**Published:** 2023-07-28

**Authors:** He Xie, Zhongwen Li, Chengchao Wu, Yitian Zhao, Chengmin Lin, Zhouqian Wang, Chenxi Wang, Qinyi Gu, Minye Wang, Qinxiang Zheng, Jiewei Jiang, Wei Chen

**Affiliations:** ^1^ National Clinical Research Center for Ocular Diseases, Eye Hospital, Wenzhou Medical University, Wenzhou, China; ^2^ Ningbo Eye Hospital, Wenzhou Medical University, Ningbo, China; ^3^ School of Electronic Engineering, Xi’an University of Posts and Telecommunications, Xi’an, China; ^4^ Cixi Institute of Biomedical Engineering, Ningbo Institute of Materials Technology and Engineering, Chinese Academy of Sciences, Ningbo, China; ^5^ Department of Ophthalmology, Wenzhou Hospital of Integrated Traditional Chinese and Western Medicine, Wenzhou, China

**Keywords:** artificial intelligence, deep learning, visual impairment, cataracts, fundus images

## Abstract

**Purpose:** To develop a visual function-based deep learning system (DLS) using fundus images to screen for visually impaired cataracts.

**Materials and methods:** A total of 8,395 fundus images (5,245 subjects) with corresponding visual function parameters collected from three clinical centers were used to develop and evaluate a DLS for classifying non-cataracts, mild cataracts, and visually impaired cataracts. Three deep learning algorithms (DenseNet121, Inception V3, and ResNet50) were leveraged to train models to obtain the best one for the system. The performance of the system was evaluated using the area under the receiver operating characteristic curve (AUC), sensitivity, and specificity.

**Results:** The AUC of the best algorithm (DenseNet121) on the internal test dataset and the two external test datasets were 0.998 (95% CI, 0.996–0.999) to 0.999 (95% CI, 0.998–1.000),0.938 (95% CI, 0.924–0.951) to 0.966 (95% CI, 0.946–0.983) and 0.937 (95% CI, 0.918–0.953) to 0.977 (95% CI, 0.962–0.989), respectively. In the comparison between the system and cataract specialists, better performance was observed in the system for detecting visually impaired cataracts (*p* < 0.05).

**Conclusion:** Our study shows the potential of a function-focused screening tool to identify visually impaired cataracts from fundus images, enabling timely patient referral to tertiary eye hospitals.

## 1 Introduction

Worldwide, the incidence of visual impairment is increasing (GBD, 2019 Blindness and Vision Impairment Collaborators, 2021), which is an important public health problem, with cataracts being the leading cause of visual impairment (Flaxman et al., 2017). According to recent research, among the 2.2 billion people who suffer from visual impairment worldwide, 134 million are blind, and 571 million have moderate-to-severe visual impairment in 2020 due to cataracts (Bourne et al., 2017; Flaxman et al., 2017). In low- and middle-income countries, especially in Southeast Asia and Africa, cataracts lead to higher rates of visual impairment than in high-income countries due to limited healthcare and financial resources (Lam et al., 2015). The World Health Organization (WHO) has adopted a 30 percent increase in effective coverage of cataract surgery as a new global target for eye care by 2030 (WHO, 2021). Therefore, there is an urgent need to facilitate and expedite cataract screening capabilities, especially for underserved populations.

Traditional cataract screening requires a professional ophthalmologist to assess the lens through a slit-lamp microscope (Gali et al., 2019) and grading methods based on the lens opacity classification system LOCS II (Chylack et al., 1989) or LOCS III (Chylack et al., 1993) (Lens Opacities Classification System, LOCS) and Wisconsin cataract grading system (Wong et al., 2013), which limits the efficiency of large-scale cataract screening. A simple and effective model for screening and referral remains a key challenge for the sustainable implementation of cataract screening programs. To enhance community screening for retinal disease in some countries (Lian et al., 2016; Verbraak et al., 2019), they have implemented telemedicine or artificial intelligence analysis of fundus images acquired by non-specialists. Grading the assessment of cataracts by fundus images may also be an effective solution. Abdul-Rahman used Fourier analysis to quantify optical degradation in fundus images, which was shown to be correlated well with the LOCS III (Abdul-Rahman et al., 2008).

Several studies have developed deep learning systems (DLSs) to grade the severity of cataracts based on the blurriness of fundus images. According to the visibility of the optic disk or retinal vessels of the fundus images, they classified cataracts into 3, four or 5 grades (Xiong et al., 2017; Zhang et al., 2019; Xu et al., 2020; Yue Zhou and Li, 2020). Considering that visual acuity is one of the most common indicators for evaluating the impact of cataracts on patients, it would be more meaningful to establish a visual function-based cataract grading system (WHO, 2020). This functional cataract screening program is more targeted for cataract patients, which can reduce the excessive referral of people with mild visual impairment and reduce the pressure on tertiary eye hospitals.

In this study, we developed a visual function-based DLS for populations based on fundus images, especially for the screening of visually impaired cataracts. In addition, we used images taken by different types of fundus cameras from three institutions to evaluate the effectiveness and generalizability of the system.

## 2 Materials and Methods

### 2.1 Image datasets

In this retrospectively study, a total of 6,997 fundus images (4,346 subjects) collected from Zhejiang Eye Hospital at Wenzhou (ZEHWZ) between September 2020 and March 2021 were used to develop the DLS. The ZEHWZ dataset included cataract patients whose best corrected decimal visual acuity (BCDVA) was good (>0.6) within 1 month after cataract surgery and non-cataract patients without refractive media opacities. The fundus images were captured without mydriasis before surgery. The exclusion criteria were traumatic cataracts, congenital cataracts and lens dislocation, corneal diseases, asteroid hyalosis, vitreous haemorrhage, and severe retinal and optic nerve diseases. Poor quality and unreadable images were also excluded: images out of focus; images underexposed; images overexposed; incomplete images with more than 1/3 peripheral halo.

Two additional datasets, including 1,398 fundus images obtained from two other institutions retrospectively, adopted the same inclusion criteria and exclusion criteria as ZEHWZ for external testing. One was derived from the inpatient department at Zhejiang Eye Hospital at Hangzhou (ZEHHZ), consisting of 1,097 images from 730 individuals; the other was derived from outpatient clinics and the inpatient department at Ningbo Eye Hospital (NEH), consisting of 301 images from 169 individuals.

This study adhered to the principles of the Declaration of Helsinki and was approved by the Ethics Committee of Zhejiang Eye Hospital at Wenzhou (Number, 2022-008-K-06-01). Due to the retrospective study design and the use of fully anonymized fundus images, the need for informed patient consent was waived by the review committee.

### 2.2 Criteria of cataract classification

The diagnosis of each fundus image was diagnosed by two cataract specialists based on the previous medical records and the results of the ophthalmology examination. If there was a difference between the two cataract specialists, there would be a third senior cataract specialists for diagnosis. All fundus images with a definitive diagnosis were screened for quality control. Poor quality and unrecognizable images were excluded.

**FIGURE 1:**
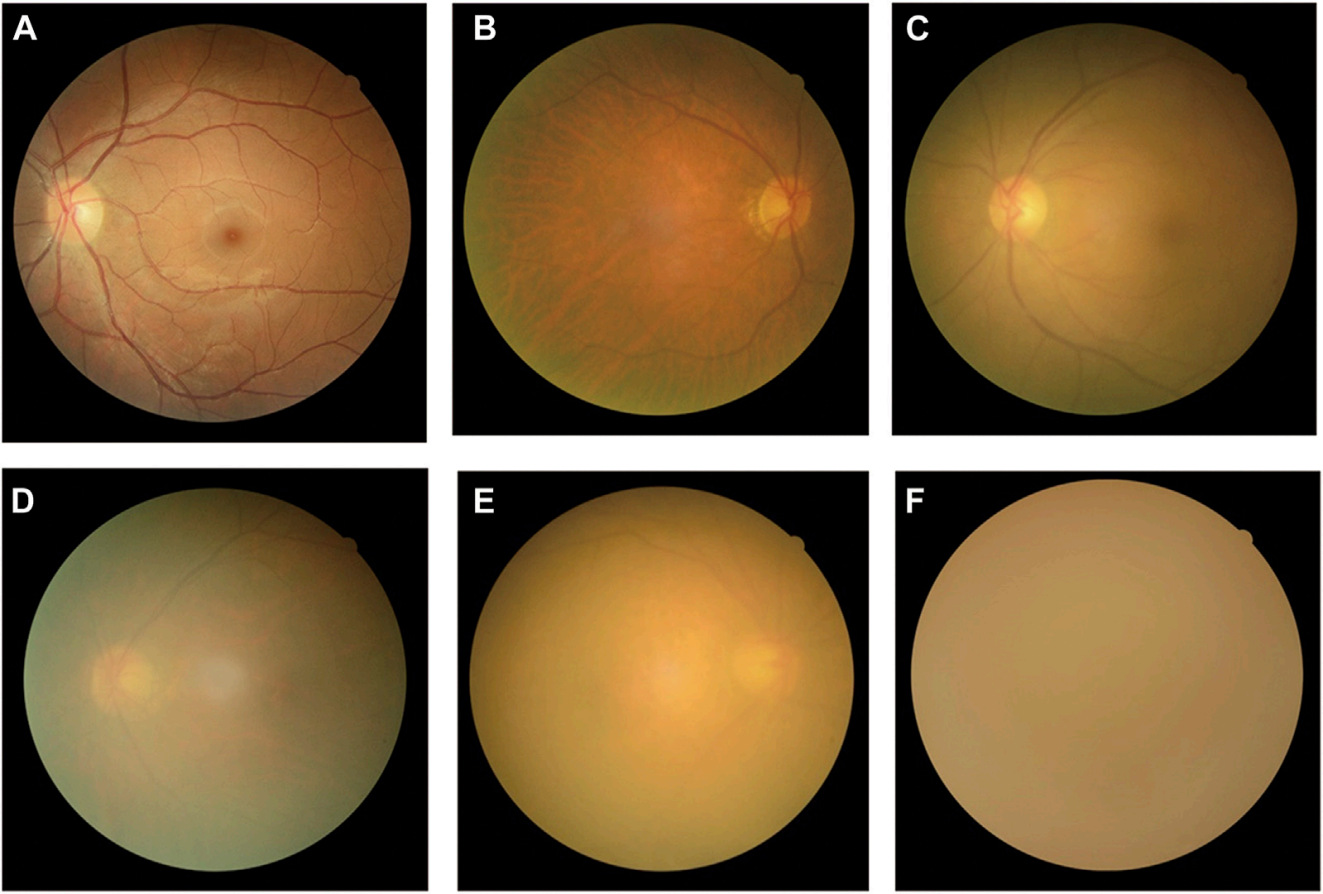
Typical examples of fundus images of non-cataracts, mild cataracts, and visually impaired cataracts **(A)** Non-cataracts **(B)** The cataract with BCDVA = 0.8 **(C)** The cataract with BCDVA = 0.5 **(D)** The cataract with BCDVA = 0.3 **(E)** The cataract with BCDVA = 0.1 **(F)** The cataract with BCDVA = HM/BE.

All fundus images were classified into three categories: non-cataracts, mild cataracts, and visually impaired cataracts. Non-cataracts were defined as patients with transparent lenses and without refractive media opacities. Mild cataracts were defined as cataracts with mild vision impairment with BCDVA ≥0.3, and visually impaired cataracts were defined as cataracts with moderate-to-severe vision impairment or blindness with BCDVA < 0.3. Typical examples of non-cataract and cataract fundus images are displayed in Figure 1.

### 2.3 Image preprocessing

During image preprocessing, each image was uniformly scaled down to 224 × 224 pixels, and the pixel values were normalized between 0 and 1. Then, data augmentation techniques were applied to increase the diversity of the dataset and thereby alleviate the overfitting problem during deep learning training. The new samples were generated by a simple transformation of the original image, simulating “real world” acquisition conditions. Random cropping, rotation of 90°, and horizontal and vertical flipping were applied to the images of the training dataset to increase the sample size to six times the original size (from 4,901 to 29,406).

### 2.4 Development and evaluation of the DLS

The fundus images drawn from the ZEHWZ dataset were randomly divided into training, validation, and internal test datasets at a ratio of 70%:15%:15%. The training and validation datasets were used to develop the system, and the test dataset was used to evaluate the performance of the system. Images from the same person were only assigned to a single dataset to prevent deep learning leaks and biased evaluations.

To find the best deep learning model for distinguishing non-cataracts, mild cataracts, and visually impaired cataracts, three convolutional neural network (CNN) architectures (DenseNet121, Inception-v3, and ResNet50) were compared. The parameters of the CNN were initialized with weights pretrained for ImageNet classification.

The deep learning models were trained using PyTorch (version 1.6.0) as the backend. Using the Adaptive Estimation of Moments (ADAM) optimizer, the initial learning rate was 0.001, β1 was 0.9, β2 was 0.999, and the weight decay was 1e-4. Each model was trained for 80 epochs. During the training, the validation loss was evaluated on the validation dataset after each epoch and used as a reference for model selection. Each time the validation loss was reduced, the model state and corresponding weight matrix were saved. The model state with the lowest validation loss was saved as the final state of the model for the test dataset.

**FIGURE 2:**
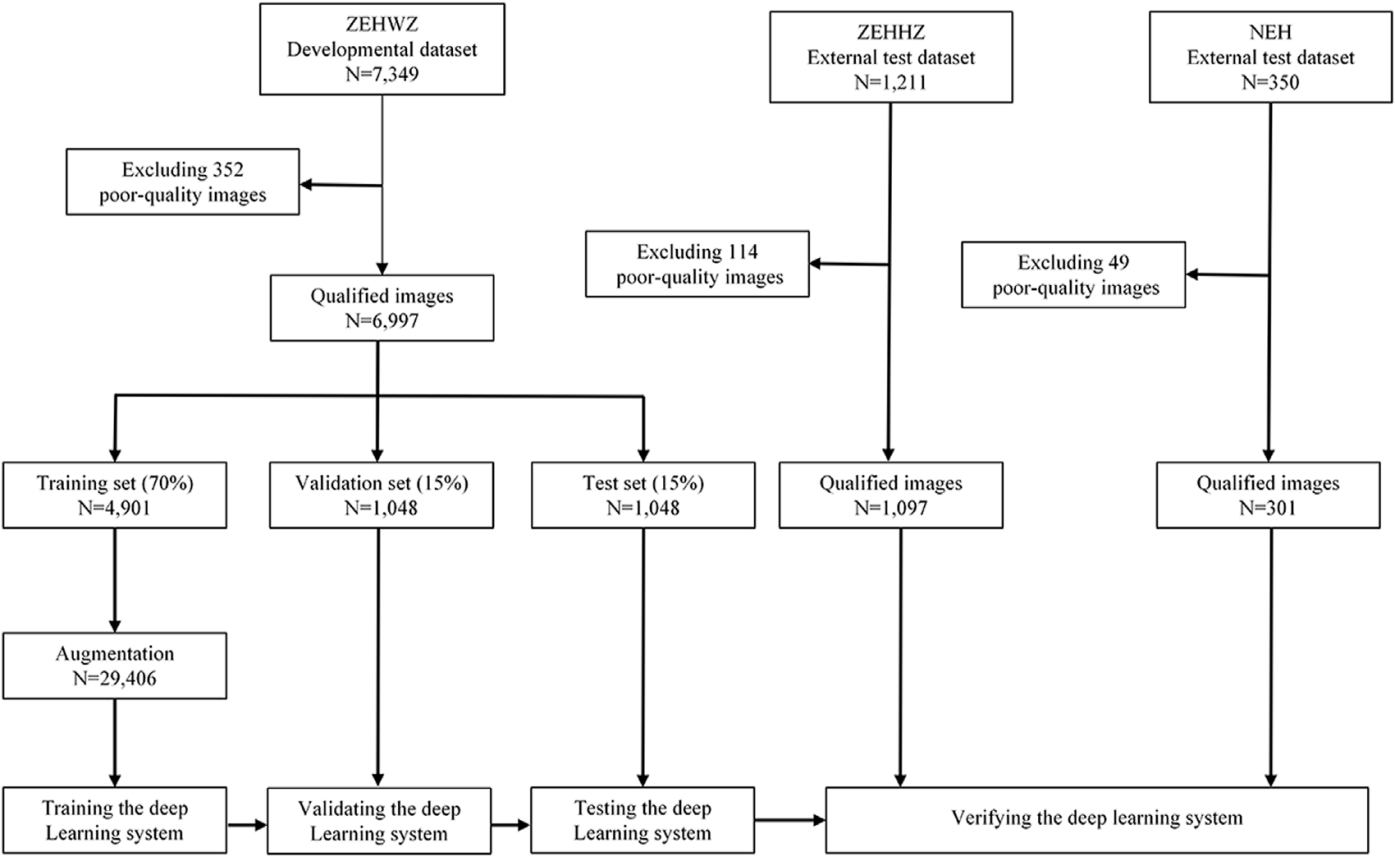
Flow chart for the development and evaluation of the DLS. ZEHWZ = Zhejiang Eye Hospital at Wenzhou; ZEHHZ = Zhejiang Eye Hospital at Hangzhou; NEH = Ningbo Eye Hospital.

The diagnostic performance of the three-class classification model was then evaluated on two independent external test datasets. The development and evaluation process of the system is shown in Figure 2. Using the t-distributed stochastic neighbour embedding (t-SNE) technique, the embedding features of each class learned by the model were displayed in a two-dimensional space.

### 2.5 Visualization heatmap

To understand which areas of fundus images were most likely to be used by deep learning models to generate decisions for this system, we use the Gradient-weighted Class Activation Mapping (GradCAM) technique to generate heatmaps. This technique uses the gradients of any target concept, flowing into the final convolutional layer to produce a localization map highlighting the important regions in the image for predicting the concept (Ramprasaath et al., 2020). Hotter colours represent the regions with more contribution to the predicted output, while cooler colours may indicate relatively less contribution to the predicted output. Using this method, heatmaps were generated to illustrate the basic principles of DLSs in differentiating between non-cataracts, mild cataracts, and visually impaired cataracts.

### 2.6 Characteristics of misclassification by the deep learning system

A senior cataract specialists who had not been involved in the initial diagnosis reviewed the characteristics of all images misclassified by the DenseNet121 algorithm and analysed the possible causes of misclassification in combination with the corresponding BCDVA.

### 2.7 DLS *versus* cataract specialists

To assess our DLS in the context of cataract detection, we recruited two cataract specialists with 3 and 10 years of clinical experience. The ZEHHZ dataset was employed to compare the performance of the best system (DenseNet121) to that of the cataract specialists with the reference standard. The system and specialists independently classified each image into one of the following three categories: non-cataracts, mild cataracts, and visually impaired cataracts. Notably, to reflect the level of experience of the cataract specialists in normal clinical practice, they were not told that they were competing with an AI-based system to avoid competition bias.

### 2.8 Statistical analysis

The performance of the deep learning system for the classification of non-cataracts, mild cataracts, and visually impaired cataracts was evaluated by employing the one-versus-rest tactic and calculating the AUC, sensitivity, specificity, and accuracy. Statistical analysis was performed using Python 3.7.8 (Wilmington, Delaware, United States of America). The 95% confidence intervals (CIs) for sensitivity, specificity, and accuracy were calculated by the Wilson scoring method using the Stats model package (version 0.11.1), and those for the area under the receiver operating characteristic (ROC) curve (AUC) were calculated using an empirical bootstrap procedure with 1,000 repetitions. We plotted the receiver operating characteristic (ROC) curve to demonstrate the capability of the system by plotting the ratio of true positive cases (sensitivity) to false positive cases (1-specificity) using the Scikit-learn (version 0.23.2) and Matplotlib (version 3.3.1) packages; a larger AUC indicated better performance. Unweighted Cohen’s kappa coefficients were calculated to compare the results of the system to a reference standard. Differences in sensitivity, specificity, and accuracy between systems and the cataract specialists were analysed using the McNemar test. All statistical tests were two-sided with a significance level of 0.05.

## 3 Results

### 3.1 Characteristics of the datasets

After removing 515 poor-quality images, a total of 8,395 qualified images (3,569 images of non-cataracts, 3,245 images of mild cataracts, and 1,581 images of visually impaired cataracts) from 5,245 individuals were used to develop and externally evaluate the DLS. Further information on the datasets from ZEHWZ, ZEHHZ, and NEH is summarized in Table 1.

**TABLE 1:** Summary of datasets.

Item	ZEHWZ dataset			ZEHHZ dataset	NEH dataset
Total no. of images	7,349			1,211	350
Total no. of qualified images	6,997			1,097	301
No. of subjects	4,346			730	169
Age, mean/range (years)	46.54/5–92			50.70/3–92	48.04/4–87
No. (%) of women	2,333/53.68			425/58.22	99/58.58
Camera model	Canon CR-2 PLUS AF (Japan)			Canon CR-2 (Japan)	RetiCam 3,100 (China)
	Training Set (70%) 4,901	Validation Set (15%) 1,048	Test Set (15%) 1,048		
Non-cataracts No. (%)	2,141 (43.68)	458 (43.70)	458 (43.70)	405 (36.92)	107 (35.55)
Mild cataracts No. (%)	1808 (36.89)	387 (36.93)	387 (36.93)	560 (51.05)	103 (34.22)
Visually impaired cataracts No. (%)	952 (19.42)	203 (19.37)	203 (19.37)	132 (12.03)	91 (30.23)

ZEHWZ = zhejiang eye hospital at wenzhou; ZEHHZ = zhejiang eye hospital at hangzhou; NEH , ningbo eye hospital.

### 3.2 Performance of different deep learning algorithms on the internal test dataset

**FIGURE 3:**
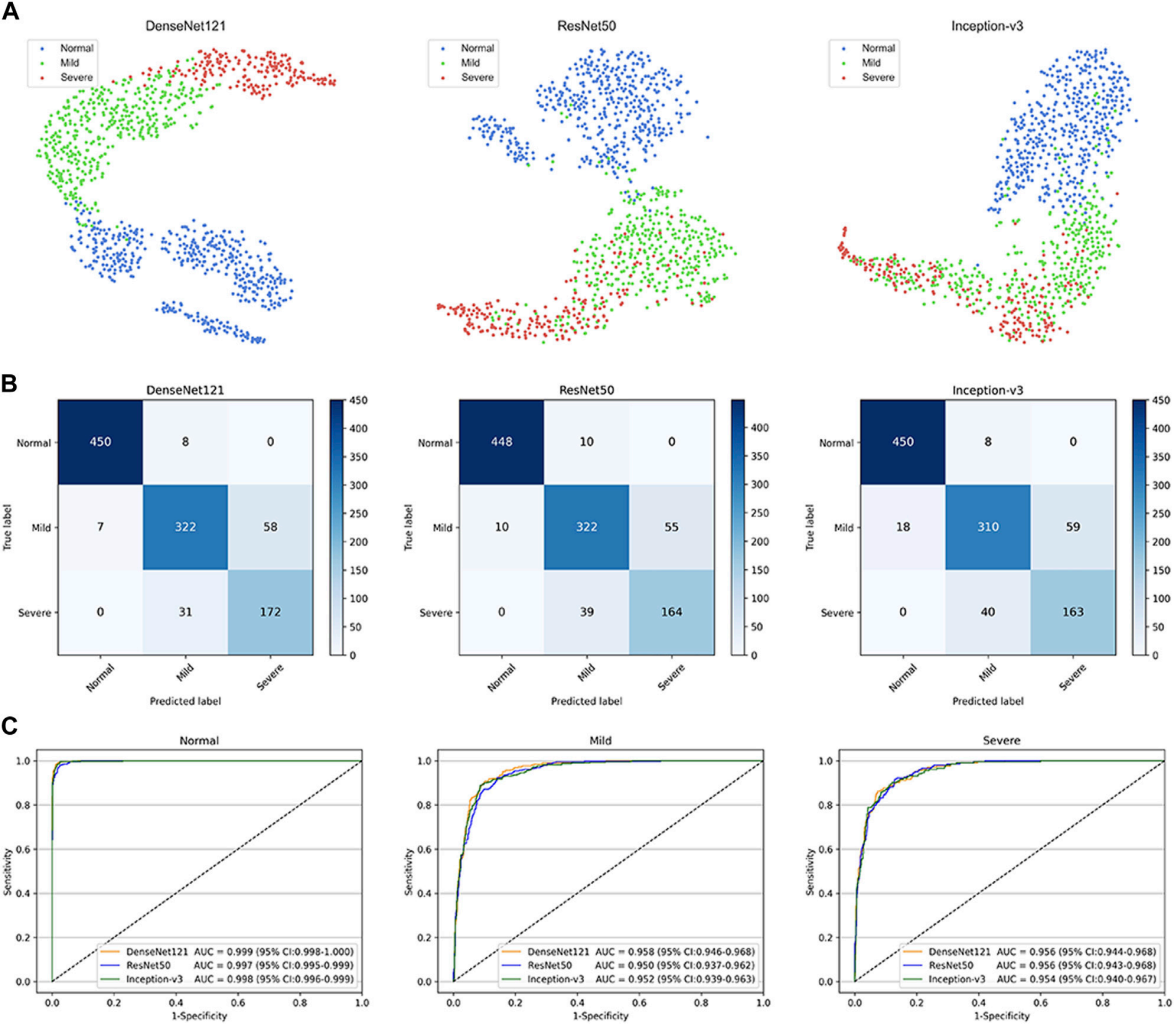
Performance of deep learning algorithms in the internal test dataset from Zhejiang Eye Hospital at Wenzhou **(A)** Visualization by t-distributed stochastic neighbour embedding (t-SNE) of the separability for the features learned by deep learning algorithms. Different coloured point clouds represent the different categories **(B)** Confusion matrices describing the accuracies of three deep learning algorithms **(C)** Receiver operating characteristic curves indicating the performance of each algorithm for detecting non-cataracts, mild cataracts, and visually impaired cataracts. “Normal” indicates non-cataracts. “Mild” indicates mild cataract. “Severe” indicates visually impairing cataract.

This study used three classical deep learning algorithms, DenseNet121, ResNet50, and Inception-v3, to train the models. The t-SNE technique showed that the features of each category learned by the DenseNet121 algorithm were more separable than those learned by ResNet50 and Inception-v3 (Figure 3A). The performance of the three algorithms on the internal test dataset is shown in Figures 3B,C, which indicates that the best algorithm was DenseNet121. More information, including the accuracy, sensitivity, and specificity of the algorithms, is presented in Table 2.

**TABLE 2:** Performance of three deep learning algorithms in the internal and external test datasets.

One-vs.-rest classification	ZEHWZ internal test dataset			ZEHHZ external test dataset			NEH external test dataset		
	Sensitivity (95% CI)	Specificity (95% CI)	Accuracy (95% CI)	Sensitivity (95% CI)	Specificity (95% CI)	Accuracy (95% CI)	Sensitivity (95% CI)	Specificity (95% CI)	Accuracy (95% CI)
Normal vs mild + severe									
DenseNet121	98.3% (97.1–99.5)	98.8% (97.9–99.7)	98.6% (97.8–99.3)	93.3% (90.9–95.8)	99.6% (99.1–100.0)	97.3% (96.3–98.2)	97.2% (94.1–100.0)	99.5% (98.5–100.0)	98.7% (97.4–100.0)
ResNet50	97.8% (96.5–99.2)	98.3% (97.3–99.3)	98.1% (97.3–98.9)	86.2% (82.8–89.5)	99.7% (99.3–100.0)	94.7% (93.4–96.0)	96.3% (92.7–99.9)	99.0% (97.5–100.0)	98.0% (96.4–99.6)
Inception-v3	98.3% (97.1–99.5)	96.9% (95.6–98.3)	97.5% (96.6–98.5)	93.1% (90.6–95.6)	96.4% (95.0–97.8)	95.2% (93.9–96.4)	94.4% (90.0–98.8)	92.3% (88.5–96.0)	93.0% (90.1–95.9)
Mild vs normal + severe									
DenseNet121	83.2% (79.5–86.9)	94.1% (92.3–95.9)	90.1% (88.3–91.9)	82.1% (79.0–85.3)	89.0% (86.4–91.7)	85.5% (83.4–87.6)	87.4% (81.0–93.8)	90.9% (86.9–94.9)	89.7% (86.3–93.1)
ResNet50	83.2% (79.5–86.9)	92.6% (90.6–94.6)	89.1% (87.2–91.0)	83.9% (80.9–87.0)	83.6% (80.5–86.7)	83.8% (81.6–86.0)	88.3% (82.2–94.5)	88.9% (84.5–93.3)	88.7% (85.1–92.3)
Inception-v3	80.1% (76.1–84.1)	92.7% (90.8–94.7)	88.1% (86.1–90.0)	80.4% (77.1–83.6)	88.5% (85.8–91.2)	84.3% (82.2–86.5)	72.8% (64.2–81.4)	88.4% (83.9–92.8)	83.1% (73.3–89.3)
Severe vs normal + mild									
DenseNet121	84.7% (79.8–89.7)	93.1% (91.4–94.8)	91.5% (89.8–93.2)	75.8% (68.4–83.1)	89.9% (88.1–91.8)	88.2% (86.3–90.1)	83.5% (75.9–91.1)	94.3% (91.1–97.4)	91.0% (87.8–94.3)
ResNet50	80.3% (74.8–85.8)	93.0% (91.3–94.7)	90.6% (88.8–92.3)	74.2% (66.8–81.7)	91.2% (89.4–93.0)	89.2% (87.3–91.0)	81.3% (73.3–89.3)	93.8% (90.6–97.1)	90.0% (86.6–93.4)
Inception-v3	80.8% (75.4–86.2)	93.5% (91.8–95.2)	91.0% (89.3–92.8)	75.8% (68.4–83.1)	90.9% (89.1–92.7)	89.1% (87.2–90.9)	80.2% (72.0–88.4)	95.2% (92.4–98.1)	90.7% (87.4–94.0)

ZEHWZ = zhejiang eye hospital at wenzhou; ZEHHZ = zhejiang eye hospital at hangzhou; NEH = ningbo eye hospital.

“Normal” indicates non-cataracts. “Mild” indicates mild cataracts. “Severe” indicates visually impaired cataracts.

The best algorithm achieved an AUC of 0.999 (95% confidence interval [CI], 0.998–1.000), a sensitivity of 98.3% (95% CI, 97.1–99.5), and a specificity of 98.8% (95% CI (97.9–99.7)) in detecting non-cataracts. The best algorithm discriminated mild cataracts from non-cataracts and visually impaired cataracts with an AUC of 0.958 (95% CI, 0.946–0.968), a sensitivity of 83.2% (95% CI, 79.5–86.9), and a specificity of 94.1% (95% CI, 92.3–95.9). The best algorithm discriminated visually impaired cataracts from non-cataracts and mild cataracts with an AUC of 0.956 (95% CI, 0.944–0.968), a sensitivity of 84.7% (95% CI, 79.8–89.7), and a specificity of 93.1% (95% CI, 91.4–94.8). Based on the reference standard of the internal test dataset, the unweighted Cohen’s kappa coefficient of the best algorithm, DenseNet121, was 0.845 (0.817–0.873).

### 3.3 Performance of the different deep learning algorithms on the external test datasets

**FIGURE 4:**
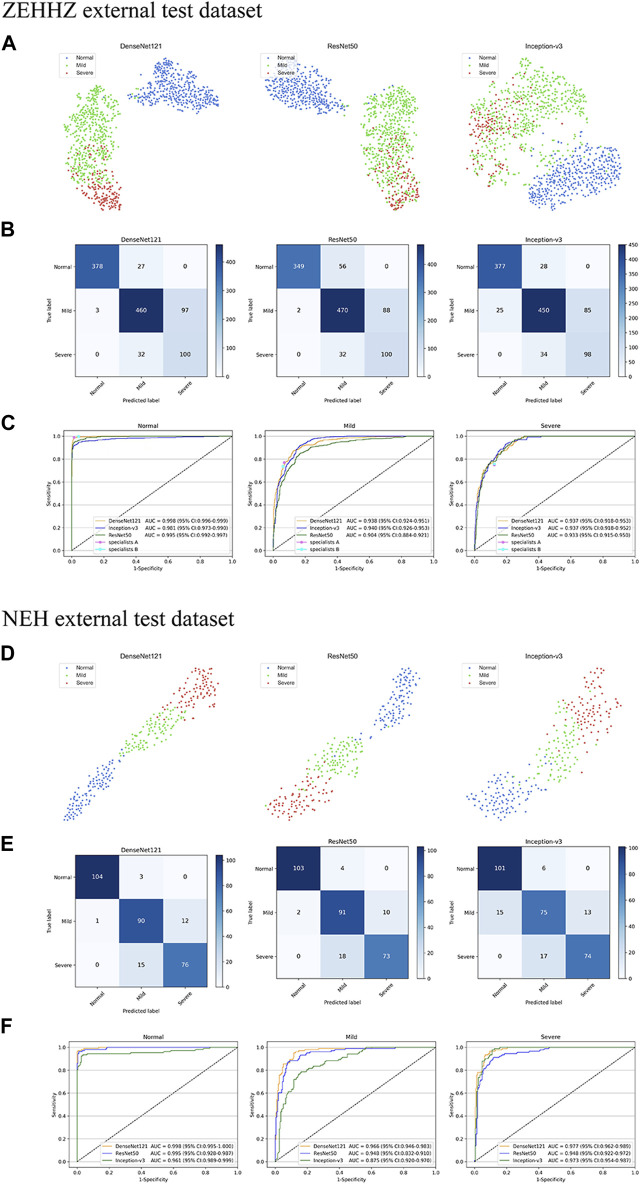
Confusion matrices and receiver operating characteristic (ROC) curves for three deep learning algorithms performance in two external test datasets. The t-distributed stochastic neighbour embedding (t-SNE) **(A–D)** presenting the separability for the features learned by deep learning algorithms in ZEHHZ and NEH external test datasets. Confusion matrices **(B–E)** describing the accuracies of two deep learning algorithms in the ZEHHZ and NEH external test datasets. ROC curves **(C–F)** indicating the performance of each algorithm for discriminating among non-cataracts, mild cataracts, and visually impaired cataracts in the ZEHHZ and NEH external test datasets. The performance of two cataract specialists were also indicated **(C)**. ZEHHZ, Zhejiang Eye Hospital at Hangzhou. NEH, Ningbo Eye Hospital. “Normal” indicates non-cataracts. “Mild” indicates mild cataract. “Severe” indicates visually impaired cataract.

The performance of the DenseNet121, ResNet50, and Inception-v3 algorithms for cataract validation on the external test dataset is shown in Figure 4, confirming that DenseNet121 achieved the best performance. The t-SNE technique also indicated that the features of each category learned by the DenseNet121 algorithm were more separable than those learned by Inception-v3 and ResNet50 (Figure 4A–D).

For the ZEHHZ dataset, the system based on DenseNet121 achieved AUCs of 0.998 (95% CI, 0.996–0.999), 0.938 (95% CI, 0.924–0.951), and 0.937 (95% CI, 0.918–0.953) in the classification of non-cataracts, mild cataracts, and visually impaired cataracts, respectively. In the NEH dataset, the system based on DenseNet121 achieved AUCs of 0.998 (95% CI, 0.995–1.000), 0.966 (95% CI, 0.946–0.983), and 0.977 (95% CI, 0.962–0.989) in the classification of non-cataracts, mild cataracts, and visually impaired cataracts, respectively.

The details on the classification performance of the three algorithms with the external datasets are shown in Table 2. In the ZEHHZ dataset, the accuracies of the best algorithm (DenseNet121) in the detection of non-cataracts, mild cataracts, and visually impaired cataracts were 97.3% (95% CI, 96.3–98.2), 85.5% (95% CI, 83.4–87.6), and 88.2% (95% CI, 86.3–90.1), respectively. In the NEH dataset, the accuracies of the best algorithm in the detection of non-cataracts, mild cataracts, and visually impaired cataracts were 98.7% (95% CI, 97.4–100.0), 89.7% (95% CI, 86.3–93.1), and 91.0% (95% CI, 87.8–94.3), respectively.

Based on the reference standards of the ZEHHZ and NEH datasets, the unweighted Cohen’s kappa coefficients of the best algorithm, DenseNet121, were 0.762 (0.728–0.796) and 0.845 (0.793–0.897), respectively.

### 3.4 Heatmaps

**FIGURE 5:**
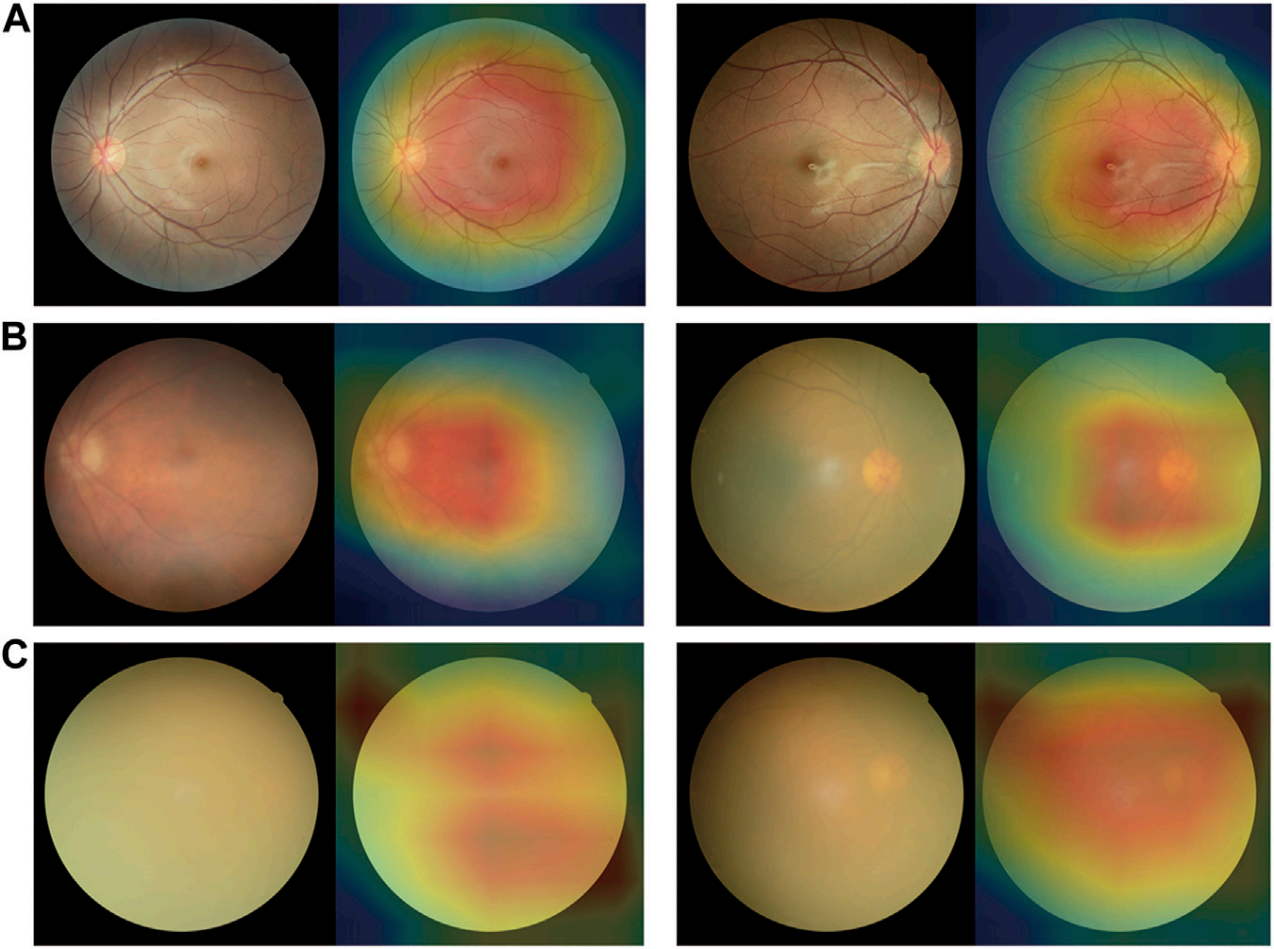
Saliency maps highlighting regions that the algorithm focuses on when making classification **(A)** Non-cataracts **(B)** mild cataracts **(C)** visually impaired cataracts. Each category is shown in a pair of an original image (left) and a corresponding heatmap (right). In these heatmaps, hotter areas (i.e., reds and oranges) are indicative of regions with increased contributions towards the predicted output, and colder regions (blues and greens) might be indicative of relatively less contribution. For each subgroup, each set of two images (from two different eyes) consistently shows the same region or feature highlighted by the algorithm.

We use heatmaps to provide insights into regions of the fundus images that might influence the algorithm’s prediction. Based on the heatmaps shown in Figure 5, we observed that the regions highlighted by the algorithm matched well with the clear features on the fundus image. For the fundus images of the non-cataracts, the region highlighted by the heatmaps was relatively consistent: large range, circular, and centred. For the fundus images of mild cataracts, the regions highlighted by the heatmaps are smaller, eccentric, oval, and around the optic disk, For the fundus images of visually impaired cataracts, the regions highlighted by the heatmaps are irregular. Figure 5 shows typical heatmaps of non-cataracts, mild cataracts, and visually impaired cataracts, respectively.

### 3.5 Classification errors

**FIGURE 6:**
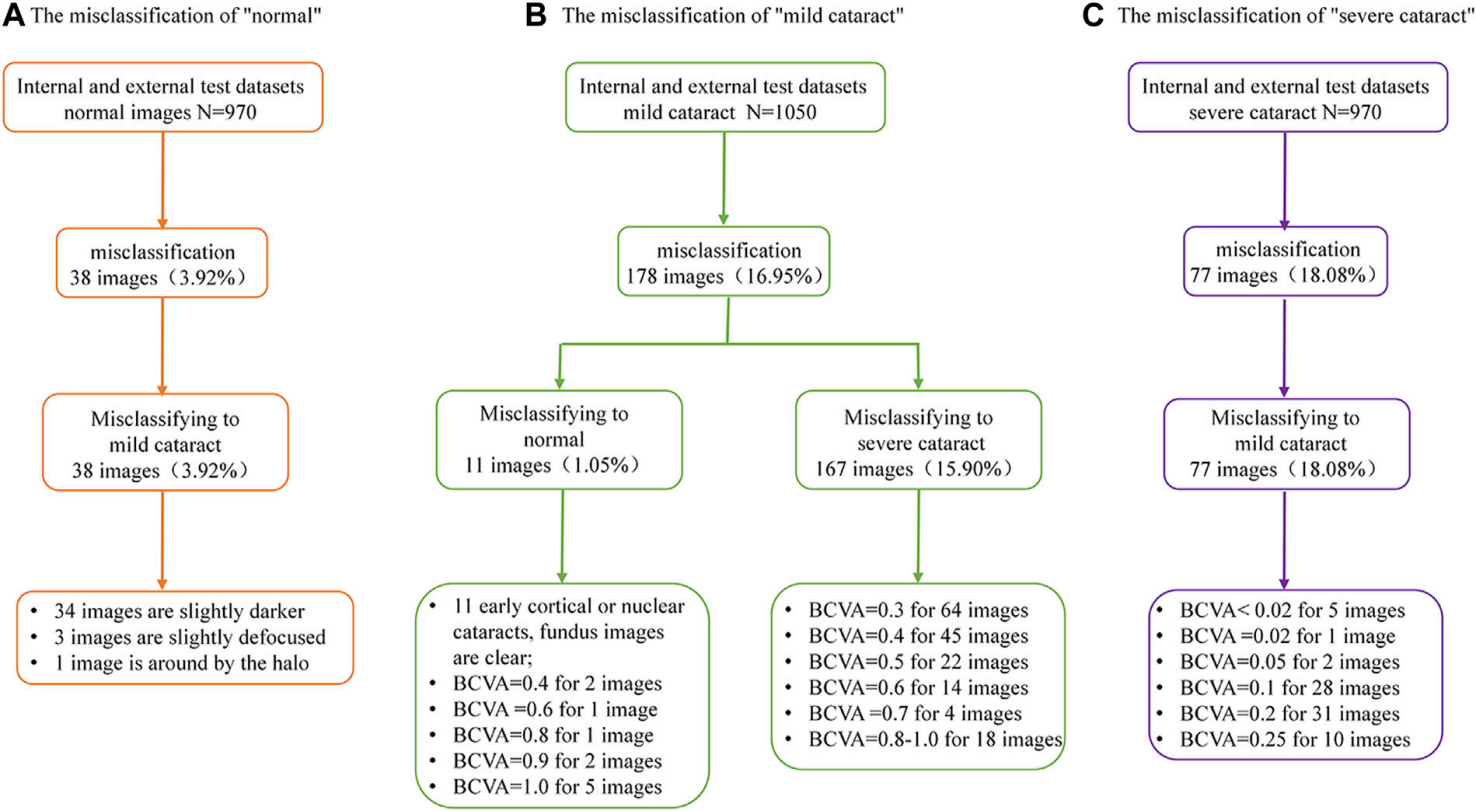
Details of deep learning system error classification in internal and external test datasets. **(A)** The misclassfication of the non-cataracts group; **(B)** The misclassfication of the mild cataracts group; **(C)** The misclassfication of the visually impaired cataracts group.

**FIGURE 7:**
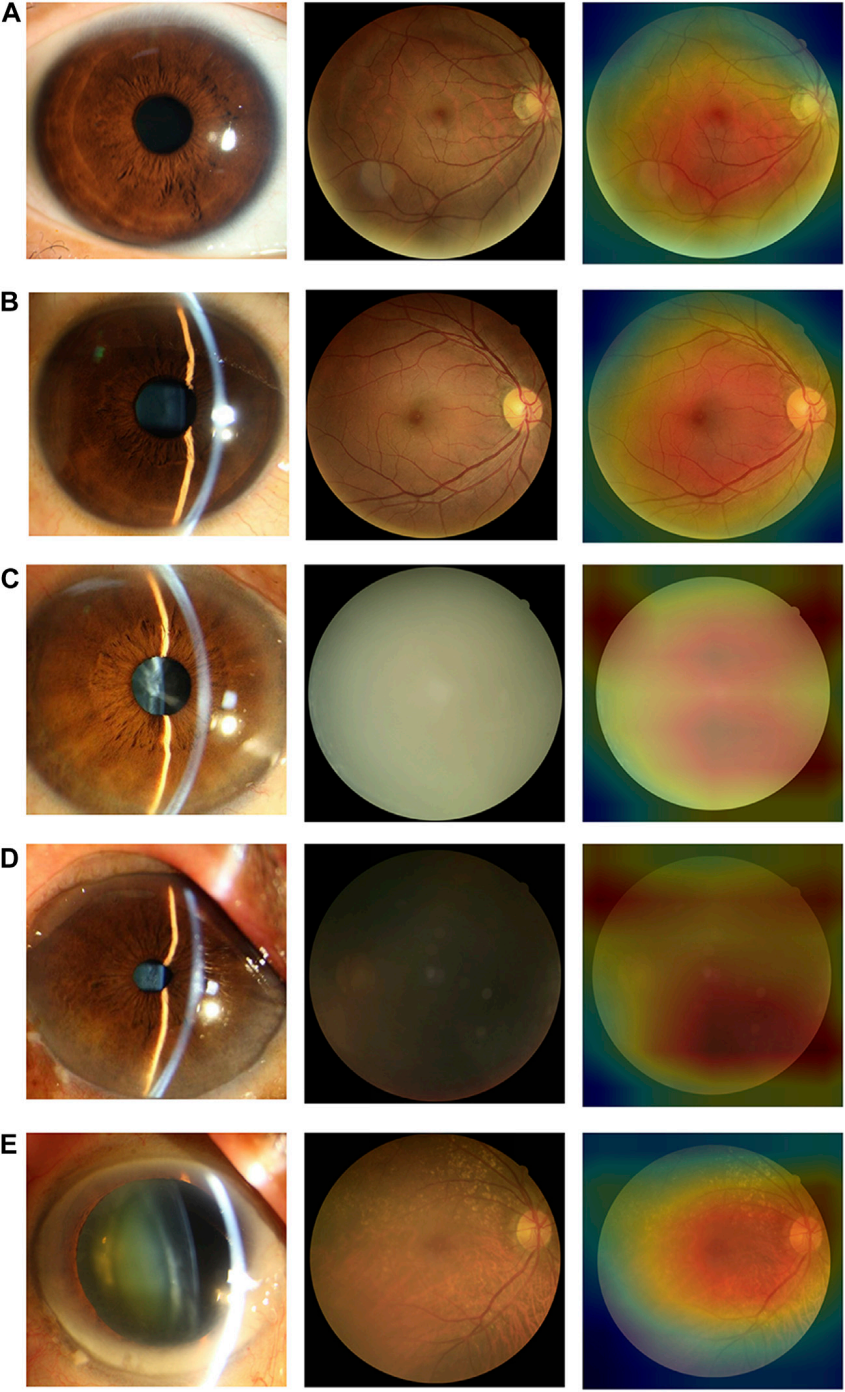
Typical examples of misclassified images by the DLS **(A)** Images of “non-cataract” incorrectly classified as “mild cataract”. The fundus image was around by the halo **(B)** Images of “mild cataract” incorrectly classified as “non-cataract”. The patient had cataracts in the early stage, BCDVA = 1.0 **(C)** Images of “mild cataract” incorrectly classified as “visually impaired cataract”. The patient had advanced cortical opacity, BCDVA = 0.6 **(D)** Images of “mild cataract” incorrectly classified as “visually impaired cataract”. Patients with small pupils reduced the amount of light entering their eyes (BCDVA = 0.4) **(E)** Images of “visually impaired cataract” incorrectly classified as “mild cataract”. The patient had a small-scale posterior subcapsular area, BCDVA = 0.16.

In the internal and external test datasets, a total of 293 images (11.98% of the total 2,446) were inconsistent with the diagnostic reference standard by the DenseNet121 algorithm. In the non-cataracts group (970 images), 38 images (3.92%) were misclassified as mild cataracts by the system, 89.47% (34 images) of which were misclassified due to dark shooting, the region highlighted by the heatmaps was eccentric and oval, as the mild cataracts, for the images were slightly darker, slightly defocused or surrounded by the halo. In the mild cataracts group (1,050 images), 11 images (1.05%) were misclassified as non-cataracts by the system due to clarity of the fundus images, most of the patients are early cortical or nuclear cataracts, the highlighted region of the heatmaps show large range, circular, and centred, as the non-cataracts. 167 (15.90%) images were misclassified as visually impaired cataracts by the system, of which 65.27% images had relatively poor BCDVA (BCDVA < 0.5) with blurred fundus images and 10.78% had good BCDVA (BCDVA between 0.8–1.0) with advanced cortical opacity, whose fundus images were blurred, the highlighted region of the heatmaps was irregular, as the visually impaired cataracts. In the visually impaired cataracts group (426 images), 77 images (18.08%) were systematically misclassified as mild cataracts, the heatmaps show the characteristic of the mild cataracts: smaller, eccentric, oval, and around the optic disk, because among these classification errors, most cataracts’ BCDVAs were not too bad (89.61% of the Images had BCDVA ≥0.1). The misclassification BCDVA situation of the DLS is shown in Figure 6. Figure 7 shows typical example of misclassified images of “non-cataract” incorrectly classified as “mild cataract”, misclassified images of “mild cataract” incorrectly classified as “non-cataract”, images of “mild cataract” incorrectly classified as “visually impaired cataract”, and images of “visually impaired cataract” incorrectly classified as “mild cataract”, respectively.

### 3.6 Comparison of the deep learning system and cataract specialists

In the ZEHHZ dataset, for the classification of non-cataracts, mild cataracts, and visually impaired cataracts, the cataract specialist with 3 years of experience achieved accuracies of 98.7% (98.1–99.4), 84.9% (82.7–87.0), and 86.1% (84.1–88.2), respectively, the senior cataract specialist with 10 years of experience achieved accuracies of 97.3% (96.3–98.2), 83.5% (81.3–85.7) and 86.2% (84.2–88.3), respectively, and the DLS achieved accuracies of 97.3% (96.3–98.2), 85.5% (83.4–87.6) and 88.2% (86.3–90.1), respectively. Our system had comparable performance to that of cataract specialists in classifying non-cataracts and mild cataracts and had better performance in classifying visually impaired cataracts (*p* < 0.05) (Table 3 and Figure 4C).

**TABLE 3:** Performance comparison of DenseNet121 with cataract specialists in the ZEHHZ dataset.

	DenseNet121	Specialists A	Specialists B	P1	P2
Normal vs mild + severe					
Sensitivity (95% CI)	93.3% (90.9–95.8)	99.0% (98.0–100.0)	99.8% (99.3–100.0)	0.000	0.000
Specificity (95% CI)	99.6% (99.1–100.0)	98.6% (97.7–99.4)	95.8% (94.3–97.3)	0.065	0.000
Accuracy (95% CI)	97.3% (96.3–98.2)	98.7% (98.1–99.4)	97.3% (96.3–98.2)	0.014	1.000
Mild vs normal + severe					
Sensitivity (95% CI)	82.1% (79.0–85.3)	77.0% (73.5–80.5)	73.6% (69.9–77.2)	0.001	0.000
Specificity (95% CI)	89.0% (86.4–91.7)	93.1% (91.0–95.3)	93.9% (91.8–95.9)	0.002	0.000
Accuracy (95% CI)	85.5% (83.4–87.6)	84.9% (82.7–87.0)	83.5% (81.3–85.7)	0.576	0.074
Severe vs normal + mild					
Sensitivity (95% CI)	75.8% (68.4–83.1)	75.0% (67.6–82.4)	75.8% (68.4–83.1)	1.000	1.000
Specificity (95% CI)	89.9% (88.1–91.8)	87.7% (85.6–89.7)	87.7% (85.6–89.7)	0.005	0.006
Accuracy (95% CI)	88.2% (86.3–90.1)	86.1% (84.1–88.2)	86.2% (84.2–88.3)	0.012	0.019

ZEHHZ = Zhejiang Eye Hospital at Hangzhou. P1 refers to the *p-value* that was calculated between the deep learning system and cataract specialist A using the two-sided McNemar test. P2 refers to the *p-value* that was calculated between the deep learning system and cataract specialist B using the two-sided McNemar test. Cataract specialist A has 3 years of clinical experience. Cataract specialist B has 10 years of clinical experience. “Normal” indicates non-cataracts. “Mild” indicates mild cataract. “Severe” indicates visually impairing cataract.

## 4 Discussion

We developed a single-modality DLS using only fundus images to detect both mild cataracts and visually impaired cataracts in the general population. Our main finding was that the system based on a convolutional neural network could discriminate among non-cataracts, mild cataracts, and visually impaired cataracts, and the DenseNet121 algorithm had the best performance. In the internal and two external test datasets, the AUCs of the system based on the best algorithm were 0.998–0.999, 0.938–0.966, and 0.937–0.977, respectively, which demonstrated the broad generalizability of our system. In addition, the unweighted Cohen’s kappa coefficients were 0.762–0.845, which showed good consistency between the outcomes of the DLS and the reference standard, further substantiating the effectiveness of our system. Moreover, our system has better performance in classifying visually impaired cataracts than cataract specialists.

The visual function-centric DLS in this study can serve as a simple, automated, and comprehensive cataract screening deployment tool. This system only needs to input fundus images and does not require other time-consuming and labour-intensive professional ophthalmic operations to obtain the severity of the patients’ cataract and the range of the best corrected visual acuity. Its simplicity can be used as an effective tool for community screening options, especially in resource-poor regions. It can not only screen for cataracts and but also can tell patients about their eye health. Moreover, visually impaired cataracts can be screened out and referred to tertiary eye hospitals.

**FIGURE 8:**
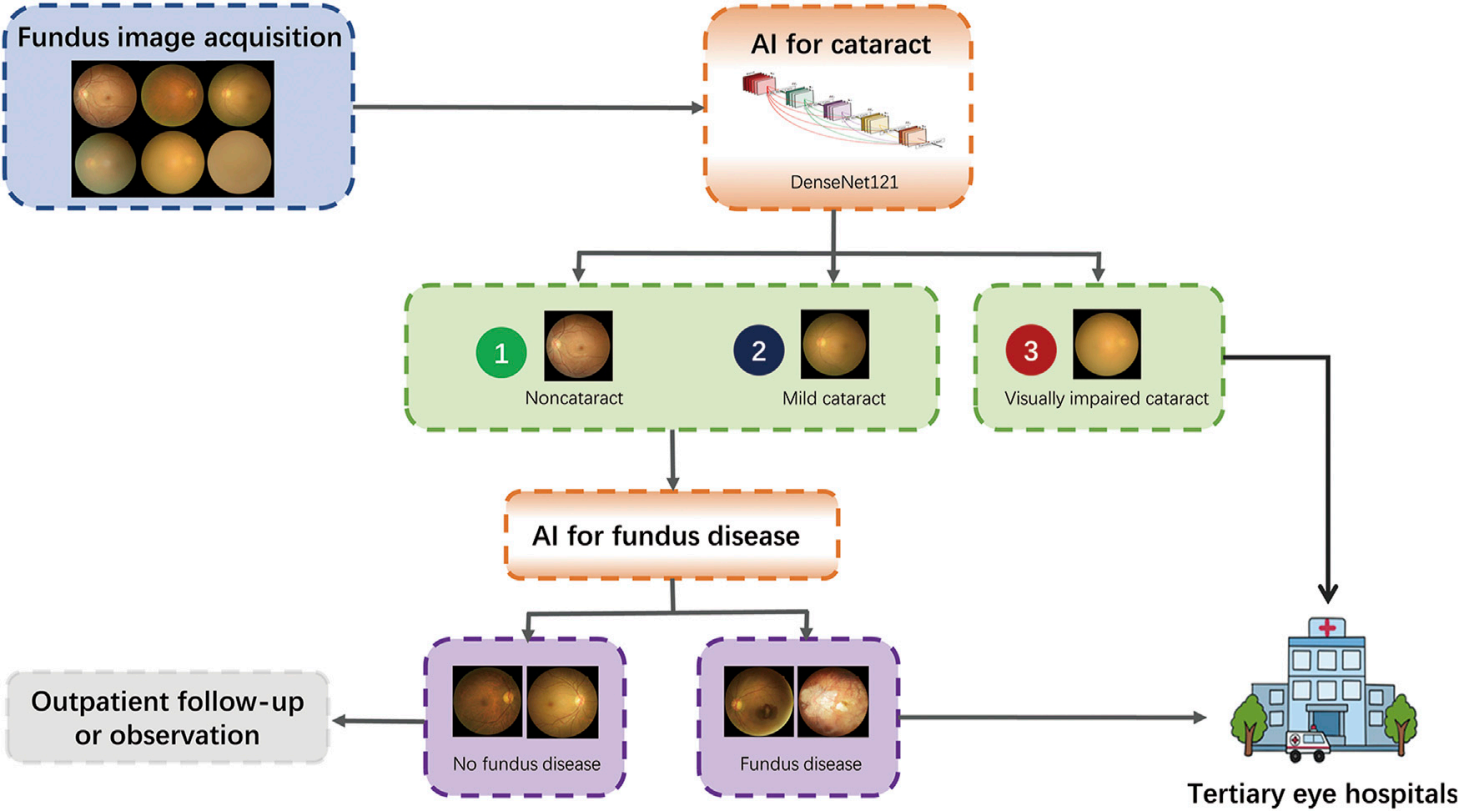
Deployment of the DLS in the existing fundus disease screening workflow.

With the increase in fundus disease-based primary care programs and community screening programs (Lin et al., 2021; Ruamviboonsuk et al., 2022), fundus photography is a routine examination procedure, and the cataract algorithm of this study can be used as an add-on algorithm to these existing devices with minimal additional cost to achieve more disease screening functions. In addition, the blurring of some fundus images caused by severe cataracts is a common cause of ungradable fundus disease (Scanlon et al., 2005). Our algorithm can screen out the fundus images of non-cataracts and mild cataracts because the fundus images of these two groups have relatively high definition, which can improve the accuracy of intelligent screening of fundus diseases and reduce the burden of unnecessary manual classification, enabling more effective referrals and improving the capacity of the existing screening programs for eye diseases. The visually impaired cataracts selected by the algorithm can be referred to a tertiary eye hospital for treatment. The workflow is shown in Figure 8.

Most of the previous studies on deep learning algorithms for cataracts based on fundus images focused on the artificial classification of the blurriness of the fundus images (Xiong et al., 2017; Zhang et al., 2019; Xu et al., 2020; Yue Zhou and Li, 2020). The annotations are subjective, and there is no accurate corresponding clinical guiding significance. In these studies, the application of these algorithms did not meet the actual situation and needs of the communities, and most of the previous studies did not consider the state of visual function. Recently, Tham et al. (2022) developed an algorithm for the automatic detection of visually significant cataracts with an AUC of 0.916–0.966. However, their algorithm can only distinguish visually significant cataracts from mild cataracts in cataract patients, but our algorithm can further classify non-cataracts from cataracts, which is of great significance for cataract screening and eye health guidance in communities. At the same time, our algorithm can also distinguish mild cataracts from non-cataracts. Although the patients only need regular follow-up and observation, we can give them some suggestions for controlling and delaying the progression of cataracts, for numerous studies had found that the risk factors for cataract formation had been associated with lifestyle and systemic diseases, include smoking, ultra-violet light exposure, alcohol intake, nutritional status, diabetes mellitus, hypertension, obesity, chronic kidney disease and autoimmune disease (Ang and Afshari, 2021). Therefore, we can advise the patients to choose a healthy lifestyle and control systemic diseases, such as controling blood sugar well. In addition, in our research, we compared three different CNN algorithms: DenseNet121, ResNet50, and Inception-v3. Among them, Densenet121 is the most accurate algorithm. It has a variety of advantages used in their study when compared to two other algorithms: alleviating the vanishing-gradient problem, strengthening feature propagation, encouraging feature reuse, and substantially improving parameter efficiency (Huang et al., 2019).

Reducing false negative misclassification of visually impaired cataracts is critical to avoid missing cataract patients who should be referred to tertiary eye centres for surgical intervention. A total of 18.08% (77/970) of visually impaired cataracts were misclassified as mild cataracts. Analysis of the misclassified fundus images found that 89.61% (69/77) of them had moderate visual impairment (0.1 ≤ BCDVA<0.3). The optometry to get BCDVA is subjective and requires the patient’s cooperation. Some cataract patients with relatively poor visual acuity might give up their efforts to see some small optotypes. Therefore, the actual visual acuity of the patients may be slightly better than the checked visual acuity. Additionally, this misclassification may be caused by a small-scale posterior subcapsular cataract. This type of cataract has a greater impact on visual acuity, while its small-scale turbidity has less impact on the quality of fundus images (Stifter et al., 2005). Reducing false positive cataract results for visually impaired cataracts is also an important consideration in community screening programs to avoid unnecessary referrals. In this study, 65.27% (109/178) of patients incorrectly diagnosed with cataracts had BCDVA < 0.5. In some countries, the population in need of cataract surgery is defined as having BCDVA<0.5, with cataracts as the main cause of vision impairment or blindness (WHO, 2021). Referral of these patients would not waste medical resources. Some patients with advanced cortical opacity have poor contrast sensitivity, although their visual acuity is good (Maraini et al., 1994). Therefore, these false positives may still need to be referred to a tertiary eye centre and cannot be completely considered incorrect referrals.

This study has several limitations. First, we did not investigate the influence of corneal diseases and vitreous haemorrhage on fundus images. However, the incidence of spontaneous vitreous haemorrhage and corneal opacity in the general population is low, 0.007% (Manuchehri and Kirkby, 2003) and 3.7% (Mukhija et al., 2020), respectively. If the patient has corneal opacity or vitreous haemorrhage, he or she must go to the hospital for further examination, and the recommendation given by the system would still apply. Second, the optometry is affected by patient compliance. Therefore, misclassification due to subjective measurement errors cannot be completely ruled out.

We developed and evaluated a novel single-modality, fundus image-based DLS for the detection of cataracts, especially visually impaired cataracts. The performance of the DLS is comparable to that of the experienced cataract specialist, indicating that this DLS can not only be used to screen cataract patients but also facilitate a timelier and more accurate referral of visually impaired cataract patients from communities to tertiary eye hospitals.

## Data Availability

The raw data supporting the conclusion of this article will be made available by the authors, without undue reservation.

## Abbreviations

DLS; Deep learning system, ROC; Receiver operating characteristic curve, WHO; World Health Organization, LOCS; Lens Opacities Classification System, ZEHWZ; Zhejiang Eye Hospital at Wenzhou, ZEHHZ; Zhejiang Eye Hospital at Hangzhou, NEH; Ningbo Eye Hospital, BCDVA; Best corrected decimal visual acuity, CNN; Convolutional neural network, ADAM; Adaptive Estimation of Moments, t-SNE; t-distributed stochastic neighbour embedding; GradCAM, Gradient-weighted Class Activation Mapping; CIs, Confidence intervals; AUC, Area under the curve.
